# A real‐world study of immune checkpoint inhibitors in advanced triple‐negative breast cancer

**DOI:** 10.1002/cai2.70

**Published:** 2023-04-23

**Authors:** Zheng Zhang, Yadi Zhang, Chuanling Liu, Jiakang Shao, Yimeng Chen, Yimin Zhu, Li Zhang, Boyu Qin, Ziqing Kong, Xixi Wang, Yutong Wang, Deqin Huang, Liqun Liu, Yuxin Zhou, Ran Tao, Zengjie Yang, Mei Liu, Weihong Zhao

**Affiliations:** ^1^ Medical School of Chinese PLA Beijing China; ^2^ Nankai University School of Medicine Tianjin China; ^3^ Department of Medical Oncology Xi'an Jiaotong University Xi'an Shaanxi China; ^4^ Department of Medical Oncology, Fifth Medical Center General Hospital of the Chinese People's Liberation Army Beijing China; ^5^ Department of Medical Oncology, First Medical Center General Hospital of the Chinese People's Liberation Army Beijing China; ^6^ Cancer Biology Program Fox Chase Cancer Center Philadelphia Pennsylvania USA; ^7^ Department of Pathology, First Medical Center General Hospital of the Chinese People's Liberation Army Beijing China

**Keywords:** biomarkers, efficacy evaluation, ICIs, prognosis, safety, TNBC

## Abstract

**Background:**

Triple‐negative breast cancer (TNBC) is the most aggressive type of breast cancer. Immune checkpoint inhibitors (ICIs) have been widely used to treat various tumors and have changed the landscape of tumor management, but the data from real‐world studies of ICIs for TNBC treatment remain limited. The aim of this study was to evaluate the efficacy of ICIs in the treatment of patients with advanced TNBC in a real‐world setting and to explore possible correlates.

**Methods:**

The clinical data of advanced TNBC patients who received ICI treatment in the Chinese People's Liberation Army (PLA) General Hospital were collected. Treatment responses, outcomes and adverse events (AEs) were assessed.

**Results:**

Eighty‐one patients were included in the study. The confirmed objective response rate (ORR) was 32.1%, and the disease control rate (DCR) was 64.2%. The median progression‐free survival (PFS) was 4.2 months, and the median overall survival (OS) was 11.0 months. PFS and OS were longer in patients who achieved clinical benefit from ICIs and shorter in patients who received later‐line ICIs and higher levels of inflammation; specifically, patients with higher TILs had longer PFS. Overall AEs were tolerable.

**Conclusions:**

ICIs are effective in the treatment of advanced TNBC, and the adverse reactions are tolerable. A panel of biomarkers including LDH, ALP, and bNLR were identified to predict the efficacies of ICIs in TNBC treatment.

## Graphical abstract

In this study, we reviewed 81 cases of advanced Triple‐negative breast cancer (TNBC) patients treated with immune checkpoint inhibitors (ICIs), and we found ICIs are effective in the treatment of advanced TNBC with tolerable adverse effects. A series of biomarkers including LDH, ALP and bNLR, are identified to predict the efficacies of ICIs in TNBC treatment.

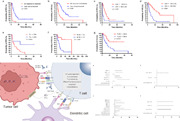

**Abbreviations**
AEs: adverse events
BMI: body mass index
CI: confidence interval
CR: complete response
CSC: cancer stem cell
DCR: disease control rate
ECOG PS: Eastern Cooperative Oncology Group performance status
FDA: Food and Drug Administration
HR: hazard ratio
ICIs: immune checkpoint inhibitors
irAEs: immune‐related adverse events
ORR: objective response rate
OS: overall survival
PD: progressive disease
PD1: programmed cell death receptor 1
PD‐L1: programmed cell death‐Ligand 1
PFS: progression‐free survival
PR: partial response
RECIST: response Evaluation Criteria in Solid Tumors
SD: stable disease
TAM: tumor‐associated macrophages
TMB: tumor mutational burden
TNBC: triple‐negative breast cancer
TRAEs: treatment‐related adverse events

## 1 INTRODUCTION

Approximately 298 000 women worldwide were diagnosed with breast cancer in 2022, accounting for 31% of the incidence of malignancies in women [1]. TNBC is a notorious subtype of breast cancer lacking estrogen receptor, progesterone receptor, and human epidermal growth factor receptor‐2 overexpression, accounting for 10%–24% of all breast cancers [2]. The classic treatment of breast cancer includes chemotherapy, endocrine therapy, targeted therapy, and so forth. Neither endocrine therapy nor targeted therapy is suitable for TNBC, so the treatment options are limited and the prognosis is poor. Patients with advanced TNBC often develop chemotherapy resistance rapidly, and the median overall survival (OS) is only 10–13 months [3]. More effective TNBC treatment strategies need to be explored. A study observes a high degree of tumor‐associated macrophage (TAM) infiltration in TNBC compared with other types of breast cancer, which may promote the invasive behavior of TNBC by enhancing epithelial mesenchymal transition (EMT) and cancer stem cell (CSC) signaling in TNBC via CCL2/Akt/beta‐catenin [4]. Along with PARP inhibitors and antibody‐drug conjugates (such as sacituzumab govitecan) for tumors associated with germline BRCA1/2 mutations, ICIs are also advancing treatment strategies for patients with advanced TNBC [5]. Tumor cells can suppress immune cells through high expression of immune checkpoint signals to evade the immune system, and ICIs can break this immune suppression. ICIs include programmed cell death receptor (PD1) and programmed cell death ligand‐1 (PD‐L1), mainly by overcoming tumor cell‐mediated immune dysfunction, destroying immune tolerance, enhancing or restoring a patient's own antitumor immunity to kill tumor cells. ICIs have made breakthroughs in the treatment of melanoma, nonsmall cell lung cancer and other tumors. Although breast cancer is less immunogenic than other solid tumors such as kidney cancer, nonsmall cell lung cancer, or melanoma that benefit from immunotherapy, there is evidence that TNBC is more immunogenic than other breast cancer subtypes with high levels of PD‐L1 expression and tumor‐infiltrating lymphocyte (TIL) penetration in the lesion [6, 7]. The results of the IMpassion130 study showed that atezolizumab combined with nab‐paclitaxel as the first‐line treatment of advanced TNBC patients significantly improved the prognosis of patients, and found that the benefit was more obvious in the PD‐L1 positive subgroup [8, 9]. However, a similarly designed study, IMpassion131, in which the chemotherapeutic agent was solvent‐based paclitaxel, did not reach equally significant conclusions. The Keynote‐355 study showed that the efficacy of pembrolizumab combined with chemotherapy tended to increase with the increase of PD‐L1 expression. Accordingly, the US Food and Drug Administration (FDA) approved pembrolizumab combined with chemotherapy in November 2020 for the treatment of locally recurrent, unresectable or metastatic TNBC patients with high PD‐L1 expression (combined positive score [CPS] ≥10) [10, 11]. Despite prolonged OS and outcomes in TNBC patients, only a small proportion of patients benefit from immunotherapy. So far, there is no established approach to predict which breast cancer patients will respond well to immunotherapy. Previous studies have found that the PD‐L1 expression levels, percentage of CD8^+^ T cells, TIL density, and tumor mutational burden (TMB) may be related to the efficacy of immunotherapy [12, 13, 14]. However, real‐world data on the application of ICIs in TNBC are still limited, and more specific biomarkers for predicting the benefit of immunotherapy in TNBC patients are unknown. Therefore, we collected clinical data such as general conditions, pathological indicators, and peripheral blood indicators of TNBC patients receiving immunotherapy in our hospital to explore the clinical factors and potential biomarkers affecting the efficacy of TNBC immunotherapy in this real‐world study.

## 2 MATERIALS AND METHODS

### 2.1 Study design and patients

#### 2.1.1 Subjects, ethical approval, and study design

The data of patients with advanced TNBC who received ICI treatment at the PLA General Hospital from January 2014 to November 2022 were collected. Inclusion criteria were: (a) patients with histologically confirmed metastatic TNBC; (b) treated with ICIs; and (c) Eastern Cooperative Oncology Group performance status (ECOG PS) ≤2. Exclusion criteria were (a) PD1/PD‐L1 inhibitors used in neoadjuvant or adjuvant therapy; (b) lack of necessary efficacy and safety evaluation data. Patients received ICIs monotherapy or ICIs combination therapy. The Ethics Committee of PLA General Hospital approved this study in compliance with the Declaration of Helsinki. The clinical data of the above patients were collected through medical record review, data entry and telephone follow‐up until December 17, 2022, and the time (in months) from the date of ICI application to the death of the patient or the deadline for ICIs was recorded for follow‐up. Breast cancer treatment efficacy was assessed according to Response Evaluation Criteria in Solid Tumors (RECIST, version 1.1), such as complete responses (CR), partial responses (PR), stable diseases (SD) or progressive disease (PD) [15]. OS refers to the time from the diagnosis of advanced breast cancer to all‐cause death; PFS refers to the time from the diagnosis of recurrence and metastasis, and treatment to the next recurrence, metastasis or death; ORR refers to the proportion of patients with CR + PR; and DCR refers to the proportion of patients with CR + PR + SD. All cases received ≥1 cycle of follow‐up, and the follow‐up result was PD or death evaluation, and the last follow‐up time was used as the outcome for those lost to follow‐up. The contents of enrollment and follow‐up included patients' clinical characteristics, molecular pathology, blood sample data before and after receiving immunotherapy, time and location of recurrence and metastasis, and survival period, and so forth.

### 2.2 Statistical analysis

Categorical variables were compared using *χ* ^2^ test or Fisher's exact test. Receiver operating characteristic curve was used to determine cut‐off value for quantitative data. Kaplan–Meier method was used to analyze PFS and OS, and stratified log‐rank test and/or Gehan‐Breslow‐Wilcoxon test were used for comparisons between different groups to assess differences at later or earlier time points, respectively. Independent predictors associated with PFS or OS were assessed using Cox proportional multivariate models, and HRs and 95% CIs were calculated, and two‐sided *p* < 0.05 were considered statistically significant. Data were analyzed using GraphPad Prism (version 7.01; GraphPad Software) and SPSS statistical software (version 26.0; SPSS, IBM Corporation).

## 3 RESULTS

### 3.1 Patient characteristics and treatment

**Figure 1:**
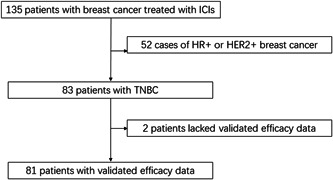
Patient inclusion and exclusion flowchart.

According to the inclusion and exclusion criteria, a total of 81 patients were included in the analysis (Figure 1). Table 1 summarizes the baseline characteristics. In brief, the median age was 51‐year‐old (range 33 to 79‐year‐old); 44 cases (54.3%) had received more than two lines of treatment, 16 cases (19.8%) with liver metastases, 12 cases (14.8%) with brain metastases, and lung metastases occurred in 35 cases (43.2%). Most patients (90.1%; *n* = 73) received combination therapy.

**Table 1:** Patient baseline characteristics.

	*n* (%)
Characteristics	Patients (*n* = 81)
*Age*	
Median (range)	51 (33–79)
<55	56 (69.1)
≥55	25 (30.9)
*ECOG PS*	
0	63 (77.8)
≥1	18 (22.2)
*BMI*	
<25 kg/m^2^	51 (63.0)
≥25 kg/m^2^	30 (37.0)
*No. of systemic treatments*	
1	37 (45.7)
≥2	44 (54.3)
*Achieving clinical benefit*	
Yes	52 (64.2)
No	29 (35.8)
*Brain metastases*	
With	12 (14.8)
Without	69 (85.2)
*Liver metastases*	
With	16 (19.8)
Without	65 (80.2)
*Pulmonary metastases*	
With	35 (43.2)
Without	46 (56.8)
*Nonvisceral metastases*	
With	68 (84.0)
Without	13 (16.0)
*Treatment group*	
ICI monotherapy	8 (9.9)
Combined treatment	73 (90.1)
*Ki‐67*	
<30%	20 (24.7)
≥30%	59 (72.8)
UNKNOWN	2 (2.5)
*TILs*	
<20%	6 (7.4)
≥20%	40 (49.4)
UNKNOWN	35 (43.2)

Abbreviations: ICI, immune checkpoint inhibitors; TILs, tumor‐infiltrating lymphocytes.

### 3.2 Efficacy

At the time of data analysis, median follow‐up was 26.3 months (range, 16.0–36.6 months); Confirmed ORR was 32.1%, DCR was 64.2%, with 2 CRs, 24 PRs and 28 SDs. The median PFS for the overall population was 4.5 months, and the median OS was 14.0 months.

### 3.3 Assessment of the relationship between clinical features and prognosis

**Figure 2:**
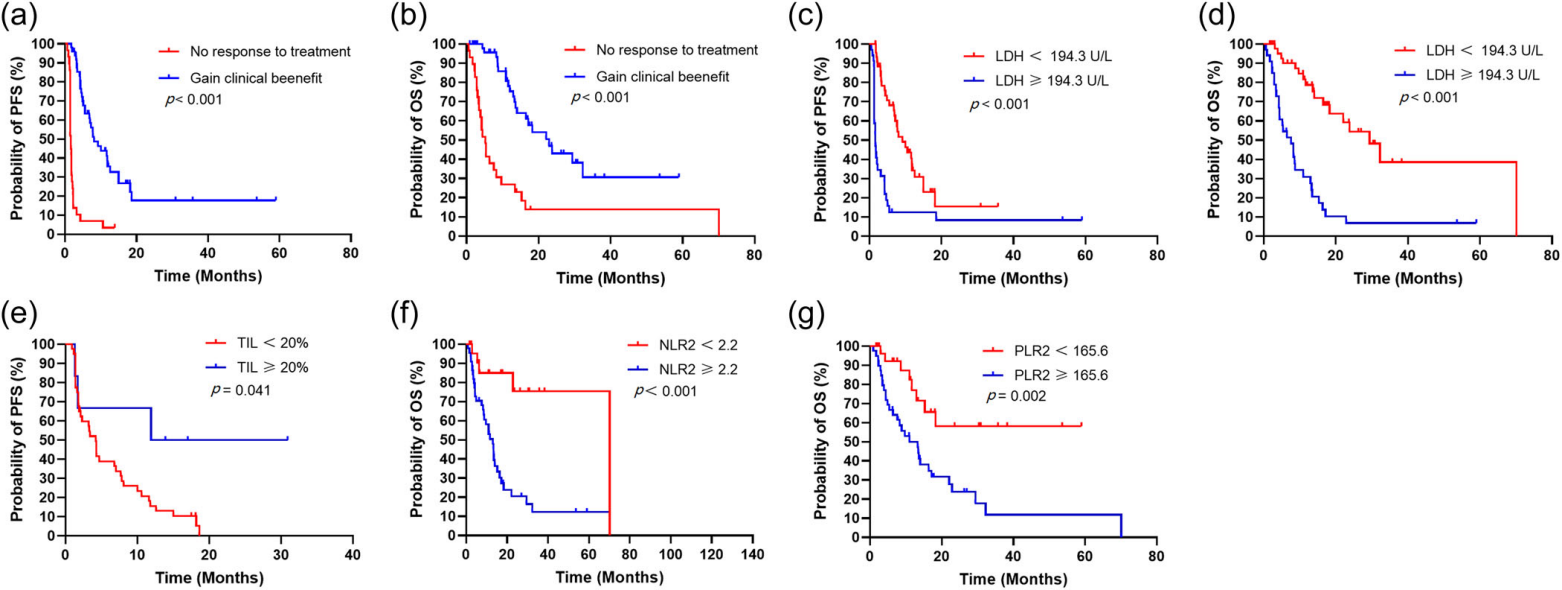
Predictive value of biomarkers identified with survival curves and log‐rank test. (a) Prognostic value of response to treatment in PFS. (b) Prognostic value of response to treatment in OS. (c) Prognostic value of LDH in PFS. (d) Prognostic value of LDH in OS. (e) Prognostic value of TILs in PFS. (f) Prognostic value of NLR2 in PFS. (g) Prognostic value of PLR2 in OS. LDH, lactate dehydrogenase; OS, overall survival; PFS, progression‐free survival.

Compared with first‐line patients, patients treated with ICIs in the second and subsequent lines tended to have shorter PFS [median, 2.3 months vs. 8.1 months; hazard ratio (HR) = 2.171; 95% confidence interval (CI): 1.296–3.636; *p* = 0.003]. Patients who achieved clinical benefit had a trend towards longer PFS (median, 8.1 months vs. 1.6 months; HR = 0.059; 95% CI: 0.028–0.126; *p* < 0.001, Figure 2a) and OS (median,22.9 months vs. 5.2 months; HR = 0.182; 95% CI: 0.090–0.366; *p* < 0.001, Figure 2b) compared to patients who did not respond to treatment. Patients with ECOG PS ≥ 1 tended to have shorter OS compared with patients with ECOG PS = 0 (median, 8.8 months vs. 16.5 months; HR = 2.421; 95% CI: 1.155–5.074; *p* = 0.019). There was a trend toward shorter OS in second‐ and higher‐line patients when ICIs were used in first‐line treatment (median, 11.0 months vs. 18.3 months; HR = 1.850; 95% CI: 1.033–3.311; *p* = 0.038). For patients with liver metastases compared with those without liver metastases (median, 8.3 months vs. 16.5 months; HR = 2.427; 95% CI: 1.072–5.495; *p* = 0.034). In addition, patients with multiple metastases tended to have shorter OS compared with patients with single metastases (median, 13.0 months vs. 23.8 months; HR = 2.154; 95% CI: 1.191–3.895; *p* = 0.011).

### 3.4 Tumor tissue biomarker assessment

In this study, we revealed a trend towards longer PFS in patients with TILs ≥20% compared with patients with TILs <20% (median, 21.4 months vs. 4.2 months; HR = 0.430; 95% CI: 0.191–0.967; *p* = 0.041, Figure 2e). However, we did not observe the correlation between Ki‐67 and immunotherapy efficacy and patient outcomes.

### 3.5 Peripheral blood biomarker assessment

Patients with pretreatment lactate dehydrogenase (LDH) ≥194.3 U/L had shorter PFS compared with patients with LDH < 194.3 U/L (median, 1.7 months vs. 9.2 months; HR = 3.889; 95% CI: 2.121–7.131; *p* < 0.001, Figure 2c). Shorter PFS was also found in patients with alkaline phosphatase (ALP) ≥104.7 U/L compared with pretreatment ALP < 104.7 U/L (median, 3.2 months vs 7.2 months; HR = 2.144; 95% CI: 1.158–3.967; *p* = 0.015), patients with a neutrophil‐to‐lymphocyte ratio (NLR) at baseline ≥2.5 compared with pretreatment baseline NLR (bNLR) <2.5 (median, 3.2 months vs. 7.8 months; HR = 1.794; 95% CI: 1.055–3.050; *p* = 0.017), patients with NLR before the second dose of immunotherapy (NLR2) ≥2.2 compared with NLR2 < 2.2 (median, 3.4 months vs. 11.9 months; HR = 2.255; 95% CI: 1.278–3.981; *p* = 0.005), and patients with platelet‐to‐lymphocyte ratio before the second immunotherapy (PLR2) ≥165.6 compared with PLR2 < 165.6 (median, 3.2 months vs. 7.8 months; HR = 2.411; 95% CI: 1.374–4.229; *p* = 0.002). Prolonged PFS was observed in patients with derived neutrophil‐to‐lymphocyte ratio (dNLR) ≤1.9 compared with patients with pretreatment dNLR >1.9 (median, 8.1 months vs. 2.2 months; HR = 0.54; 95% CI: 0.323–0.904; *p* = 0.019).

Patients with LDH ≥ 194.3 U/L tended to exhibit shorter OS compared with patients with pretreatment LDH < 194.3 U/L (median, 7.6 months vs. 29.4 months; HR = 5.647; 95% CI: 2.940–10.850; *p* < 0.001, Figure 2d). A trend toward shorter OS was also observed in patients with pretreatment ALP < 104.7 U/L compared with patients with ALP ≥ 104.7 U/L (median, 8.3 months vs. 22.1 months; HR = 3.328; 95% CI: 1.679–6.596; *p* < 0.001). Patients with platelet‐to‐lymphocyte ratio (PLR) <152.3 tended to have shorter OS than patients with PLR ≥ 152.3 (median, 8.8 months vs. 22.9 months; HR = 2.244; 95% CI: 1.219–4.132; *p* = 0.010). Patients with bNLR ≥2.5 had significantly shorter OS than those with pretreatment bNLR <2.5 (median, 8.7 months vs. 23.8 months; HR = 2.781; 95% CI: 1.525–5.073; *p* < 0.001, Figure 2f). Similarly, patients with NLR2 ≥ 2.2 had significantly shorter OS than those with NLR2 < 2.2 (median, 13.0 months vs. 70.1 months; HR = 3.498; 95% CI: 1.796–6.814; *p* < 0.001). A trend toward significantly shorter OS was observed in patients with PLR2 ≥ 165.6 compared with pretreatment PLR2 < 165.6 (median, 13.3 months vs. undefined; HR = 2.756; 95% CI: 1.439–5.278; *p* = 0.002, Figure 2g), and patients with PLRtrend (PLR2/PLR) ≥1.3 compared with those with pretreatment PLRtrend <1.3 (median, 13.5 months vs. 18.3 months; HR = 2.218; 95% CI: 1.093–4.503; *p* = 0.027). However, patients with dNLR ≤1.9 had a trend towards longer OS compared with dNLR 1.9 (median, 23.8 months vs. 8.6 months; HR = 0.314; 95% CI: 0.169–0.582; *p* < 0.001).

Combining the KM curve and the results of clinical practice, some of the above variables were included in univariate and multivariate COX analyses. The results showed that in response to treatment, LDH and TIL were independent predictors of PFS. Responses to treatment, LDH, NLR2 and PLR2 were independent predictors of OS.

### 3.6 Safety

Thirty patients (37.0%) experienced at least one treatment‐related adverse events (TRAE). Fourteen patients (17.2%) experienced potentially immunotherapy‐related adverse events (irAE), the most common of which was elevated thyroid stimulating hormone (TSH, 7.4%). One grade 5 irAE was associated with grade 4 myelosuppression and immune‐related liver injury, and one was grade 3 myelosuppression. Notably, there was one immune‐related death in our study. A 43‐year‐old patient with bilateral lung, multiple bone and soft tissue metastases who received nab‐paclitaxel plus toripalimab in third‐line therapy developed grade 4 myelosuppression, grade 4 immune‐related liver injury, grade 1 renal impairment and grade 1 pancreatitis, and died shortly after receiving one cycle of treatment.

## 4 DISCUSSION

This study analyzed 81 patients with advanced TNBC treated with ICIs in our hospital, summarized their clinical features, pathological conditions and laboratory indicators, investigated their relationships with clinical response, and explored the safety of ICIs in the treatment of advanced TNBC. In the IMpassion130 study, the median PFS and OS of atezolizumab plus nab‐paclitaxel in the first‐line treatment of locally advanced or metastatic TNBC were 7.2 months and 21.0 months, respectively. The median OS in the Keynote‐355 study of pembrolizumab plus chemotherapy as first‐line treatment in patients with locally recurrent inoperable or metastatic TNBC was 17.2 months. The median PFS and OS were relatively short for the overall population in our study (4.5 months and 14.0 months, respectively). Both clinical trials included well‐appearing first‐line patients receiving ICIs, whereas more than half of our patients received ICIs at a later stage, and some with poor general condition (ECOG = 2). TNBC is one of the worst prognostic subtypes of breast cancer due to its aggressiveness and insensitivity to chemotherapy and limited treatment options. The number of TILs in breast cancer has been identified as an important prognostic factor for improved survival in TNBC patients. The high proportion of TILs in TNBC lesions and their high immunogenicity suggest that they may be more sensitive to immunotherapy [16]. However, only a small fraction of patients can benefit from immunotherapy, and there are no clear biomarkers to predict the effectiveness of ICIs against TNBC.

We examined the patients' clinical characteristics, pathological features, and relevant laboratory indices and explored their relationship with the clinical response and overall prognosis of TNBC patients treated with ICIs. This study showed that patients with an ECOG PS score of 0 had a better prognosis than patients with an ECOG PS ≥ 1, which was consistent with clinical practice, because the lower the ECOG PS score, the better the patients' physical condition, and may be better tolerated and respond to the treatment, so there is a trend towards longer PFS and OS.

The main cause of death from breast cancer is metastatic spread, and the liver is the fourth most common site of breast cancer metastasis after lymph nodes, lungs and bones. Liver metastasis is also one of the leading causes of death in patients with advanced breast cancer. Previous studies have found that breast cancer patients with liver metastases have a relatively poorer prognosis compared with patients with lung and bone metastases [17]. The prognosis of patients with liver metastases in this study was poor, consistent with previously reported results. This finding is consistent with a previous report using a preclinical model of colorectal cancer liver metastases: mice without liver metastases responded to ICIs, whereas mice with liver metastases did not. This study also found that T‐cell clones and T‐cell diversity were reduced in tumors of breast cancer patients with liver metastases, leading to systemic immunosuppression and reduced efficacy of immunotherapy [18]. Liver metastasis as a potential negative factor for the effectiveness of immunotherapy warrants further investigation.

Previous studies have shown that PD1, PD‐L1, TILs, and TMB may be potential biomarkers related to the efficacy of immunotherapy [19, 20, 21, 22]. A French study retrospectively analyzed the correlation between TILs and clinical outcomes after immunotherapy and chemotherapy in patients with advanced nonsmall cell lung cancer (NSCLC), and the results showed that patients with high density of TILs had a lower risk of death and disease recurrence, and a higher ORR when treated with Nivolumab, while no similar phenomenon was observed in the chemotherapy group [23]. Our study associated high‐density TILs (≥20%) at baseline with prolonged PFS, but found no significant difference in OS.

LDH is a catalytic enzyme in the pathways of glycolysis and gluconeogenesis, and exists in most tissues of the body [24, 25, 26]. Cancer cells produce large amount of lactic acid to form an acidic tumor microenvironment, and local high concentration of lactic acid inhibits the function of cytotoxic T lymphocytes and reduces the release of IL‐2, interferon, perforin, and granzyme [27]. It also promotes the recruitment of myeloid‐derived suppressor cells, prevents the maturation of dendritic cells, inhibits NK cytotoxicity, suppresses T cell activation, and promotes Treg cell differentiation, thereby inhibiting innate and adaptive immunity. This immunosuppressive microenvironment also limits ICI function, all of which are associated with poorer clinical outcomes [28]. Elevated baseline LDH and poor prognosis have been observed in a variety of malignancies including small‐cell lung cancer, lymphoma and multiple myeloma, and patients with elevated baseline LDH benefited significantly less from immunotherapy than patients with normal LDH [29,30]. Our study showed that PFS and OS were significantly shortened in patients with high LDH before ICI treatment. A domestic retrospective study found that TNBC patients with ALP > 66.5 U/L before treatment had the worst disease‐free survival rate and OS (*p* < 0.001) [31], and similar to the results of the present study, PFS and OS were shorter with higher ALP.

In NSCLC patients treated with ICIs, high baseline NLR and PLR values and their derivatives were significantly associated with poorer OS and PFS [32]. This effect is achieved through various chemokines and cytokines, including transforming growth factor‐β, vascular endothelial growth factor, IL‐6, IL‐8, and so forth. Recent studies have shown that lower lymphocyte counts often reflect impaired cell‐mediated immunity and that increased TILs are associated with better treatment response and prognosis in patients with solid tumors [33].

High PLR and NLR are associated with poor outcomes in breast cancer, but the mechanisms remain unknown. A Japanese study on TNBC observed a positive correlation between PLR and TIL (*p* = 0.013). TNBC patients had different outcome patterns according to TIL and PLR, with the TIL^high^/PLR^low^ group having the lowest disease recurrence and mortality, the absence of distant metastasis and the longest overall survival, while the TILs^low^/PLR^high^ group having the shortest survival [34]. A Polish study showed that the 5‐year OS was lower in the NLR > 2.65 group compared with the NLR ≤ 2.65 group (82.5% vs. 89.6%; *p* = 0.053), and the lower OS was more pronounced in the TNBC subgroup (70.3% vs. 89.3%; *p* = 0.034). Patients with a PLR > 190.9 had a lower 5‐year OS than those with a PLR ≤ 190.9 group (78.7% vs. 89.4%; *p* = 0.020). A poorer OS rate associated with elevated PLR was also observed in the TNBC subgroup (68.2% vs. 88.5%; *p* = 0.032) [35]. Consistent with these reports, our study also showed that higher bNLR and PLR were associated with poorer PFS and OS. dNLR is a novel biomarker associated with inflammation and immune response, and its role in predicting clinical outcomes has been extensively explored. In NSCLC patients treated with ICIs, high dNLR was associated with poorer PFS (HR = 1.56; *p* < 0.0001) and OS (HR = 2.02; *p* < 0.0001) [36]. A study including 308 patients with early/locally advanced TNBC explored the relationship between dNLR and pCR and found that a high dNLR at baseline might be associated with a lower pCR rate [37]. Similarly, this study found that patients with lower dNLR had longer PFS and OS. A Spanish study examined bNLR, NLR2, and NLR trend (NLR2/bNLR) and explored the association with PFS and OS. A total of 211 patients were included in the study, the low bNLR group had significantly longer PFS (HR = 0.71; 95% CI: 0.60–0.84) and OS (HR = 0.66; 95% CI: 0.55–0.79) than those of the high bNLR group, the low NLR2 group had significantly longer PFS (HR = 0.67; 95% CI: 0.57–0.79) and OS (HR = 0.60; 95% CI: 0.50–0.72) than the high NLR2 group, and finally, patients with NLRtrend <1 had significantly longer PFS (HR = 0.59; 95% CI: 0.43–0.82) and OS (HR = 0.63; 95% CI: 0.44–0.90) than patients with NLRtrend ≥1. In the multivariate analysis of PFS and OS, bNLR, NLR2 and NLRtrend were all independent prognostic factors for PFS and OS [38]. Similarly, we found that patients with higher NLR2 had shorter PFS and OS, but found no correlation between NLRtrend and PFS and OS. It is suggested that the systemic inflammatory state may be associated with the effectiveness and prognosis of immunotherapy in TNBC patients, but the specific mechanism by which the systemic inflammatory state affects the benefit of immunotherapy is still unknown.

Several noteworthy points about our study, including (a) A prospective clinical trial would greatly complement our current retrospective study; (b) A study with a larger sample size could further strengthen our conclusions from a relatively limited number of samples; (c) Future research needs to determine whether the levels of laboratory indicators such as bNLR will change when ICIs are used in combination with chemotherapy or targeted therapy; and (d) There are still some patients who have not achieved the study endpoints and need continuous follow‐up. Nevertheless, our study evaluated TNBC patients after ICI treatment from a real‐world perspective, incorporating various factors such as clinical characteristics, pathological features, and laboratory indices, providing valuable information for future immunotherapy of TNBC.

## 5 CONCLUSION

In conclusion, we found that patients with advanced TNBC had significantly shorter PFS and OS than those treated with ICIs. Some factors, such as ECOG PS score, achieving clinical benefit after treatment, liver metastasis and multiple metastases may be associated with immunotherapy in TNBC; while TIL, LDH, ALP, bNLR, PLR, dNLR, NLR2, PLR2, and PLR trend may be potential biomarkers for immunotherapy.

## AUTHOR CONTRIBUTIONS

**Zheng Zhang**: Conceptualization (lead); data curation (lead); formal analysis (lead); investigation (lead); methodology (lead); validation (lead); visualization (lead); writing—original draft (lead); writing—review & editing (equal). **Yadi Zhang**: Data curation (equal); formal analysis (equal); software (equal). **Chuanling Liu**: Data curation (equal); formal analysis (equal). **Jiakang Shao**: Investigation (equal); visualization (equal). **Yimeng Chen**: Investigation (equal); visualization (equal). **Yimin Zhu**: Investigation (equal); visualization (equal). **Li Zhang**: Investigation (equal); visualization (equal). **Boyu Qin**: Investigation (equal); visualization (equal). **Ziqing Kong**: Data curation (equal). **Xixi Wang**: Data curation (equal). **Yutong Wang**: Data curation (equal). **Deqin Huang**: Data curation (equal). **Liqun Liu**: Data curation (equal). **Yuxin Zhou**: Data curation (equal). **Ran Tao**: Data curation (equal). **Zengjie Yang**: Writing—review & editing (equal). **Mei Liu**: Writing—review & editing (lead). **Weihong Zhao**: Funding acquisition (lead); project administration (lead); resources (lead); writing—review & editing (lead).

## CONFLICT OF INTEREST STATEMENT

The authors declare no conflict of interest.

## ETHICS STATEMENT

This study was conducted in accordance with the Declaration of Helsinki, and it was approved by the Medical Ethics Committee of the General Hospital of the Chinese People's Liberation Army (NCC2020C‐211). This study has not been published in any other journal, and all authors agree to publish this study in Cancer Innovation.

## INFORMED CONSENT

Not applicable.

## Data Availability

The data that support the findings of this study are available from the corresponding author upon request.

## References

[1] Siegel RL , Miller KD , Wagle NS , Jemal A . Cancer statistics, 2023. CA Cancer J Clin. 2023;73(1):17–48. 10.3322/caac.21763 36633525

[2] Hong H , Li C , Gong L , Wang J , Li D , Shi J , et al. Universal endogenous antibody recruiting nanobodies capable of triggering immune effectors for targeted cancer immunotherapy. Chem Sci. 2021;12(12):4623–30. 10.1039/d0sc05332e 34163726 PMC8179521

[3] Waks AG , Winer EP . Breast cancer treatment. JAMA. 2019;321(3):288. 10.1001/jama.2018.19323 30667503

[4] Chen X , Yang M , Yin J , Li P , Zeng S , Zheng G , et al. Tumor‐associated macrophages promote epithelial–mesenchymal transition and the cancer stem cell properties in triple‐negative breast cancer through CCL2/AKT/β‐catenin signaling. Cell Commun Signaling. 2022;20(1):92. 10.1186/s12964-022-00888-2 PMC9205034 35715860

[5] Bagegni NA , Davis AA , Clifton KK , Ademuyiwa FO . Targeted treatment for high‐risk early‐stage triple‐negative breast cancer: spotlight on pembrolizumab. Breast Cancer: Targets Ther. 2022;14:113–23. 10.2147/BCTT.S293597 PMC9064451 35515356

[6] Mediratta K , El‐Sahli S , D'Costa V , D'Costa V , Wang L . Current progresses and challenges of immunotherapy in triple‐negative breast cancer. Cancers. 2020;12(12):3529. 10.3390/cancers12123529 33256070 PMC7761500

[7] Ribeiro R , Carvalho MJ , Goncalves J , Moreira JN . Immunotherapy in triple‐negative breast cancer: insights into tumor immune landscape and therapeutic opportunities. Front Mol Biosci. 2022;9:903065. 10.3389/fmolb.2022.903065 36060249 PMC9437219

[8] Schmid P , Rugo HS , Adams S , Schneeweiss A , Barrios CH , Iwata H , et al. Atezolizumab plus nab‐paclitaxel as first‐line treatment for unresectable, locally advanced or metastatic triple‐negative breast cancer (IMpassion130): updated efficacy results from a randomised, double‐blind, placebo‐controlled, phase 3 trial. Lancet Oncol. 2020;21(1):44–59. 10.1016/S1470-2045(19)30689-8 31786121

[9] Schmid P , Adams S , Rugo HS , Schneeweiss A , Barrios CH , Iwata H , et al. Atezolizumab and nab‐paclitaxel in advanced triple‐negative breast cancer. N Engl J Med. 2018;379(22):2108–21. 10.1056/NEJMoa1809615 30345906

[10] Cortes J , Cescon DW , Rugo HS , Nowecki Z , Im SA , Yusof MM , et al. Pembrolizumab plus chemotherapy versus placebo plus chemotherapy for previously untreated locally recurrent inoperable or metastatic triple‐negative breast cancer (KEYNOTE‐355): a randomised, placebo‐controlled, double‐blind, phase 3 clinical trial. Lancet. 2020;396(10265):1817–28. 10.1016/S0140-6736(20)32531-9 33278935

[11] Cortes J , Rugo HS , Cescon DW , Im SA , Yusof MM , Gallardo C , et al. Pembrolizumab plus chemotherapy in advanced triple‐negative breast cancer. N Engl J Med. 2022;387(3):217–26. 10.1056/NEJMoa2202809 35857659

[12] Park S , Ock CY , Kim H , Pereira S , Park S , Ma M , et al. Artificial intelligence–powered spatial analysis of tumor‐infiltrating lymphocytes as complementary biomarker for immune checkpoint inhibition in non–small‐cell lung cancer. J Clin Oncol. 2022;40(17):1916–28. 10.1200/JCO.21.02010 35271299 PMC9177249

[13] Jardim DL , Goodman A , de Melo Gagliato D , Kurzrock R . The challenges of tumor mutational burden as an immunotherapy biomarker. Cancer Cell. 2021;39(2):154–73. 10.1016/j.ccell.2020.10.001 33125859 PMC7878292

[14] Kist De Ruijter L , van de Donk PP , Hooiveld‐Noeken JS , Giesen D , Elias SG , Lub‐de Hooge MN , et al. Whole‐body CD8+ T cell visualization before and during cancer immunotherapy: a phase 1/2 trial. Nature Med. 2022;28(12):2601–10. 10.1038/s41591-022-02084-8 36471036 PMC9800278

[15] Schwartz LH , Litière S , de Vries E , Ford R , Gwyther S , Mandrekar S , et al. RECIST 1.1‐Update and clarification: from the RECIST committee. Eur J Cancer. 2016;62:132–7. 10.1016/j.ejca.2016.03.081 27189322 PMC5737828

[16] Savas P , Salgado R , Denkert C , Sotiriou C , Darcy PK , Smyth MJ , et al. Clinical relevance of host immunity in breast cancer: from TILs to the clinic. Nat Rev Clin Oncol. 2016;13(4):228–41. 10.1038/nrclinonc.2015.215 26667975

[17] Giovinazzo F , Bucaro A , Agnes S . Breast cancer liver metastasis: time to resection and criteria. Hepatobiliary Surg Nutr. 2022;11(5):749–51. 10.21037/hbsn-22-372 36268235 PMC9577979

[18] Yu J , Green MD , Li S , Sun Y , Journey SN , Choi JE , et al. Liver metastasis restrains immunotherapy efficacy via macrophage‐mediated T cell elimination. Nature Med. 2021;27(1):152–64. 10.1038/s41591-020-1131-x 33398162 PMC8095049

[19] Chen L , Huang S , Liu Q , Kong X , Su Z , Zhu M , et al. PD‐L1 protein expression is associated with good clinical outcomes and nomogram for prediction of disease free survival and overall survival in breast cancer patients received neoadjuvant chemotherapy. Front Immunol. 2022;13:849468. 10.3389/fimmu.2022.849468 35669769 PMC9163312

[20] Dai Y , Zhao W , Yue L , Dai X , Rong D , Wu F , et al. Perspectives on immunotherapy of metastatic colorectal cancer. Front Oncol. 2021;11:659964. 10.3389/fonc.2021.659964 34178645 PMC8219967

[21] Yazaki S , Salgado R , Shimoi T , Yoshida M , Shiino S , Kaneda T , et al. Impact of adjuvant chemotherapy and radiotherapy on tumour‐infiltrating lymphocytes and PD‐L1 expression in metastatic breast cancer. Br J Cancer. 2023;128(4):568–75. 10.1038/s41416-022-02072-2 36522476 PMC9938235

[22] Li J , Zhang S , Chen S , Yuan Y , Zuo M , Li T , et al. Lipid metabolism‐related gene signature predicts prognosis and depicts tumor microenvironment immune landscape in gliomas. Front Immunol. 2023;14:1021678. 10.3389/fimmu.2023.1021678 36860853 PMC9968762

[23] Gataa I , Mezquita L , Rossoni C , Auclin E , Kossai M , Aboubakar F , et al. Tumour‐infiltrating lymphocyte density is associated with favourable outcome in patients with advanced non‐small cell lung cancer treated with immunotherapy. Eur J Cancer. 2021;145:221–9. 10.1016/j.ejca.2020.10.017 33516050

[24] Liu S , Wu M , Wang F . Research progress in prognostic factors and biomarkers of ovarian cancer. J Cancer. 2021;12(13):3976–96. 10.7150/jca.47695 34093804 PMC8176232

[25] Long H , Hu CT , Prijatelj V , Weng CF . Antrodia cinnamomea is a potentially effective complementary medicine for adjuvant therapy against breast cancer with bone metastasis: a case report. Medicine. 2020;99(27):e20808. 10.1097/MD.0000000000020808 32629666 PMC7337546

[26] Lv Z , Yu Y , Luo Y , Lin S , Xiang X , Mao X , et al. Long‐term survival outcomes of pediatric adrenal malignancies: an analysis with the upstaged SEER registry during 2000‐2019. Front Endocrinol. 2022;13:977105. 10.3389/fendo.2022.977105 PMC9511147 36171902

[27] Wang Y , Jia A , Bi Y , Wang Y , Liu G . Metabolic regulation of myeloid‐derived suppressor cell function in cancer. Cells. 2020;9(4):1011. 10.3390/cells9041011 32325683 PMC7226088

[28] Pérez‐Tomás R , Pérez‐Guillén I . Lactate in the tumor microenvironment: an essential molecule in cancer progression and treatment. Cancers. 2020;12(11):3244. 10.3390/cancers12113244 33153193 PMC7693872

[29] Robert C , Long GV , Brady B , Dutriaux C , Maio M , Mortier L , et al. Nivolumab in previously untreated melanoma without BRAF mutation. N Engl J Med. 2015;372(4):320–30. 10.1056/NEJMoa1412082 25399552

[30] Robert C , Grob JJ , Stroyakovskiy D , Karaszewska B , Hauschild A , Levchenko E , et al. Five‐year outcomes with dabrafenib plus trametinib in metastatic melanoma. N Engl J Med. 2019;381(7):626–36. 10.1056/NEJMoa1904059 31166680

[31] Chen B , Dai D , Tang H , Chen X , Ai X , Huang X , et al. Pre‐treatment serum alkaline phosphatase and lactate dehydrogenase as prognostic factors in triple negative breast cancer. J Cancer. 2016;7(15):2309–16. 10.7150/jca.16622 27994669 PMC5166542

[32] Russo A , Franchina T , Ricciardi GRR , Battaglia A , Scimone A , Berenato R , et al. Baseline neutrophilia, derived neutrophil‐to‐lymphocyte ratio (dNLR), platelet‐to‐lymphocyte ratio (PLR), and outcome in non‐small cell lung cancer (NSCLC) treated with Nivolumab or Docetaxel. J Cell Physiol. 2018;233(10):6337–43. 10.1002/jcp.26609 PMC6767577 29672849

[33] Amer HT , Stein U , El Tayebi HM . The monocyte, a maestro in the tumor microenvironment (TME) of breast cancer. Cancers. 2022;14(21):5460. 10.3390/cancers14215460 36358879 PMC9658645

[34] Onagi H , Horimoto Y , Sakaguchi A , Ikarashi D , Yanagisawa N , Nakayama T , et al. High platelet‐to‐lymphocyte ratios in triple‐negative breast cancer associates with immunosuppressive status of TILs. Breast Cancer Res. 2022;24(1):67. 10.1186/s13058-022-01563-7 36217150 PMC9552414

[35] Huszno J , Kolosza Z . Prognostic value of the neutrophil‑lymphocyte, platelet‑lymphocyte and monocyte‑lymphocyte ratio in breast cancer patients. Oncol Lett. 2019;18(6):6275–83. 10.3892/ol.2019.10966 31788105 PMC6865674

[36] Mezquita L , Preeshagul I , Auclin E , Saravia D , Hendriks L , Rizvi H , et al. Predicting immunotherapy outcomes under therapy in patients with advanced NSCLC using dNLR and its early dynamics. Eur J Cancer. 2021;151:211–20. 10.1016/j.ejca.2021.03.011 34022698

[37] Ocaña A , Chacón JI , Calvo L , Antón A , Mansutti M , Albanell J , et al. Derived neutrophil‐to‐lymphocyte ratio predicts pathological complete response to neoadjuvant chemotherapy in breast cancer. Front Oncol. 2022;11:827625. 10.3389/fonc.2021.827625 35223459 PMC8875201

[38] Viñal D , Gutierrez‐Sainz L , Martinez D , Garcia‐Cuesta JA , Pedregosa J , Villamayor J , et al. Prognostic value of neutrophil‐to‐lymphocyte ratio in advanced cancer patients receiving immunotherapy. Clin Transl Oncol. 2021;23(6):1185–92. 10.1007/s12094-020-02509-1 33226553

